# Supplementary figures and images for: Learning to select actions shapes recurrent dynamics in the corticostriatal system
Source: Neural Netw. 2020 Dec;132:375–93. doi: 10.1016/j.neunet.2020.09.008 (PMC7685243; doi:10.1016/j.neunet.2020.09.008)

**A**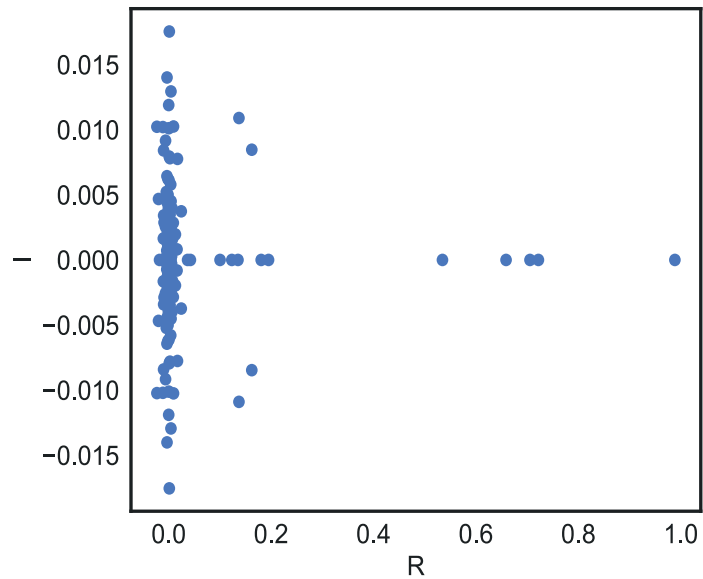**B**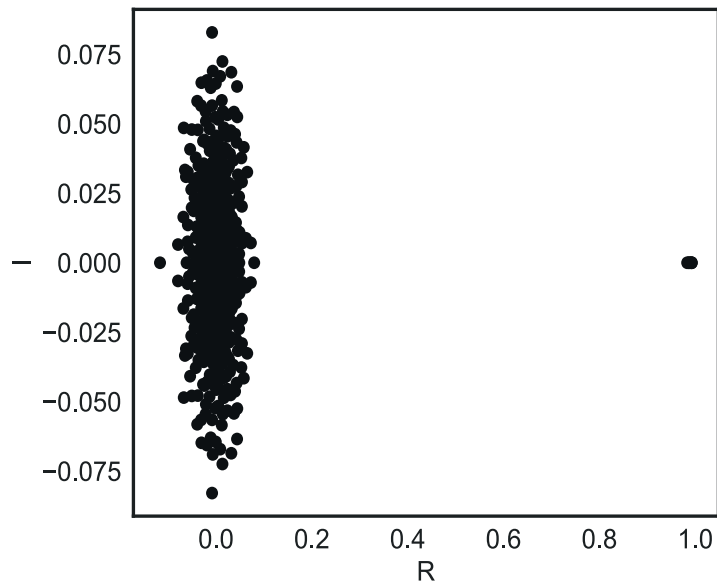

Supplement: MMC S1 — Eigenvalue spectra of the network system after training.A PFC model network, B STR model network. [file mmc1.pdf]

- S1 (**R**-U-L)
- S1 Error (**L**-U-L)
- S2 (**L**-U-R)
- S3 (**L**-D-R)
- S5 (**R**-U-R)
- S6 (**L**-U-L)
- S6 Error (**R**-U-L)
- S8 (**R**-D-R)
- S8 Error (**L**-D-R)

Trial 1, Move 1

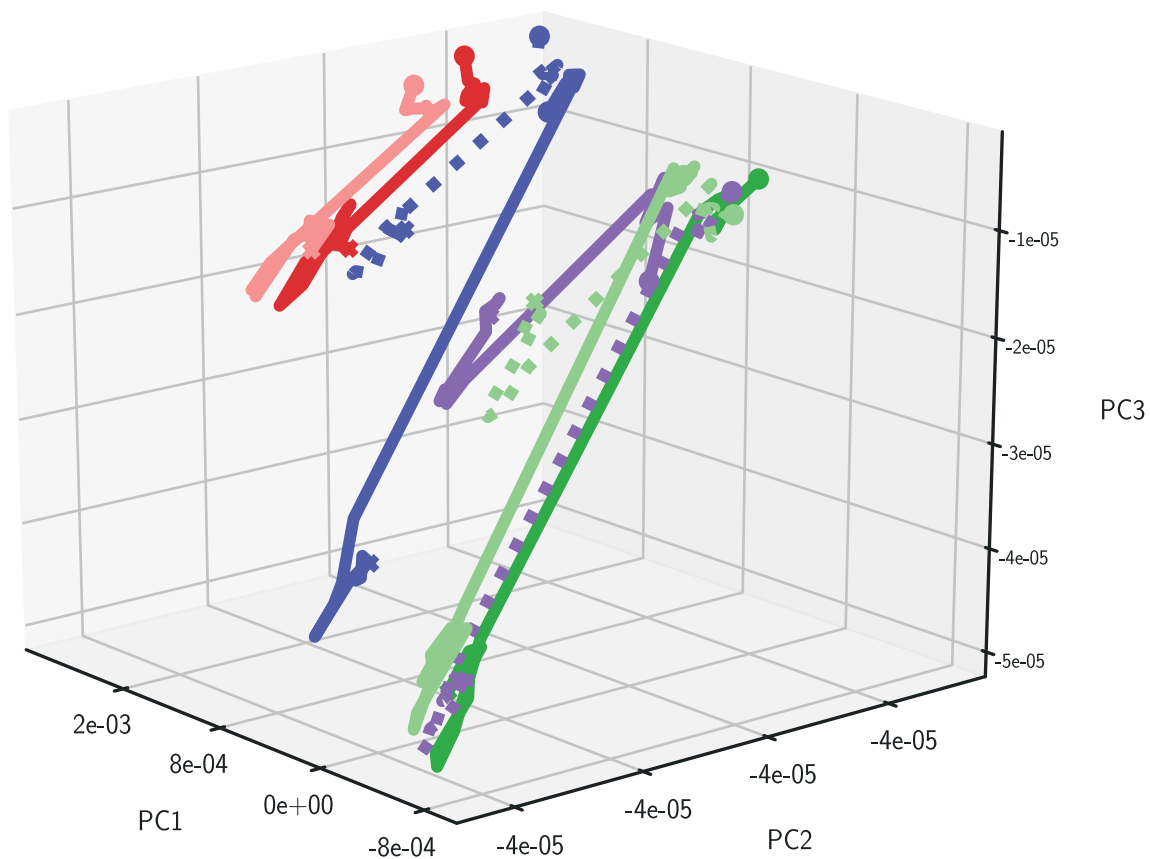

Supplement: MMC S2 — Latent representations of correct and wrong movements in the STR model network. Single movement trajectories depicted in three-dimensional state space (spanned by axes obtained through dPCA). The trajectories show the first movement in a sequence from the first trial after a sequence switch. Representations for correctly executed sequences (solid lines) are depicted alongside sequences that contained mistakes (dotted lines). [file mmc2.pdf]
